# Case Report: A Delayed Diagnosis of Congenital Syphilis—Too Many Missed Opportunities

**DOI:** 10.3389/fped.2020.499534

**Published:** 2021-01-20

**Authors:** Sarah Khafaja, Yolla Youssef, Nidale Darjani, Nour Youssef, Captain Mohammad Fattah, Rima Hanna-Wakim

**Affiliations:** ^1^Department of Pediatrics and Adolescent Medicine, American University of Beirut Medical Center, Beirut, Lebanon; ^2^Division of Pediatric Infectious Diseases, Department of Pediatrics and Adolescent Medicine, American University of Beirut Medical Center, Beirut, Lebanon; ^3^Center for Infectious Diseases Research, American University of Beirut Medical Center, Beirut, Lebanon; ^4^Military Hospital, Badaro, Beirut, Lebanon

**Keywords:** congenital syphilis, delayed diagnosis, awareness, prevention, maternal screening

## Abstract

Congenital syphilis remains a significant public health problem nowadays. We describe the presentation of an infant with a delayed diagnosis of congenital syphilis, with a negative initial non-treponemal test. Our aim is to shed light on the incidence of missed prevention, the importance of awareness, maternal screening, and early diagnosis.

## Background

Congenital syphilis remains a significant public health problem worldwide despite the availability of preventive strategies for the identification of the at-risk newborn (1). According to the World Health Organization (WHO) 2018 report on the global sexually transmitted infections (STI) surveillance, the incidence of congenital syphilis per 100.000 live births decreased from 539 in 2012 to 473 in 2016 (2, 3). Despite the global decrease in its incidence; congenital syphilis remains in a significant problem in both developing and developed countries. In the United States, the Centers for Disease Control and Prevention (CDC) reports that the number of congenital syphilis cases has significantly increased from 9.2 cases per 100.000 live births in 2013 to 23.2 cases per 100.000 live births in 2017 (4). In Lebanon, we estimate that many cases of congenital syphilis cases are underreported. Data from the epidemiological surveillance unit at the Lebanese Ministry of Public Health showed an increase in the reported cases of syphilis from 10 cases in 2002 to 21 cases in 2018 (5).

Congenital syphilis is caused by *Treponema pallidum subspecies pallidum*, which can be transmitted transplacentally to the fetus following maternal spirochetemia or intrapartum by contact with maternal genital lesions. It is classified as early congenital syphilis when the clinical manifestations occur below 2 years of age, and as late congenital syphilis when the signs and symptoms appear after the 2nd year (6). Timely identification and treatment of pregnant women early in the pregnancy will decrease significantly the sequelae of congenital syphilis. In addition, early diagnosis and management of infected newborns will prevent the manifestations of late congenital syphilis. We report an infant with a delayed diagnosis of congenital syphilis as the initial non-treponemal test was non-reactive. This case sheds light on the incidence of missed prevention, the importance of awareness, maternal screening, and early diagnosis.

## Case Presentation

A 5-months-old male infant was born by cesarean delivery at term with a birth weight of 4 kg to a G3P3 mother who had an uncomplicated pregnancy. The mother had routine prenatal care with an obstetrician-gynecologist; she had several visits during pregnancy that included prenatal screening for anemia, hepatitis B infection, rubella, and toxoplasma serology. The mother is a housewife, she has a seizure disorder, and is maintained on antiepileptic medications. The father is a governmental employee with no comorbidity. She was neither screened for syphilis nor treated during the pregnancy. At 2 months of age, the infant developed a facial and perioral rash and was noted to have poor weight gain. He had no vomiting, bloody diarrhea, irritability, or other symptoms. He was suspected to have cow's milk protein allergy and was started on extensively hydrolyzed infant formula. At 3 months of age, he developed fever associated with rhinorrhea and respiratory distress for which he was hospitalized at an outside hospital and received intravenous antibiotics (unknown) for 3 days. At around 4 and a half-month of age, he continued to have failure to thrive with a weight below 0.01% (*Z* score = −3.92) on the WHO growth chart for boys (0–2 years of age). At that time, the father mentioned to the treating physician that he was treated with doxycycline for syphilis around 1 year before presentation. He also reported having unprotected sexual intercourse with the infant's mother during the pregnancy. Subsequently, the infant had a negative serum Venereal Diseases Research Laboratory (VDRL) test but a positive treponemal antibodies (by Treponema pallidum indirect hemagglutination assay-TPHA). Blood for HIV antibodies by ELISA was non-reactive.

The mother had a reactive VDRL and positive treponemal antibodies and was started on doxycycline by her physician. The infant was referred to our hospital for further management of suspected congenital syphilis.

On physical examination, he had a scaly maculopapular salmon-colored rash on the cheeks with mild rhinitis (snuffles), hypotonia, bulging anterior fontanelle, significant head lag, bilateral nystagmus, and hypospadias. No hepatosplenomegaly or visible musculoskeletal abnormalities were noted.

The initial laboratory tests showed leukocytosis (18,600/mm^3^), anemia (hemoglobin, 8.4 g/dl), and thrombocytosis (664,000/mm^3^). The rapid plasma reagin test (RPR) was positive at a dilution of 1:256, and the TPHA also was reactive at 1:20480, both of which were 4-fold higher than the respective maternal titers (RPR 1:32; TPHA 1:2560). Cerebrospinal fluid (CSF) studies showed CSF WBC 5/mm^3^, CSF RBC 350/mm^3^, CSF protein not elevated (0.34 g/L), CSF glucose 43 mg/dL, and non-reactive CSF VDRL test.

Further evaluations including echocardiography, ophthalmology (no evidence of chorioretinitis, uveitis, cataract, or glaucoma), abdominal ultrasound (no hepatosplenomegaly), brain magnetic resonance imaging (MRI) with no evidence of meningeal enhancement or hydrocephalus, and auditory brain-stem response (ABR) were negative. Long bone radiography showed moderate bilateral periosteal reaction along the femoral and tibial diaphysis with no metaphyseal abnormalities which could be related to the patient's congenital syphilis.

The patient received a 10-days course of intravenous aqueous penicillin G (50,000 units/kg every 6 h) after which he was discharged home.

To note that we obtained an informed consent from the infant's father for publication of this case report.

## Discussion

Congenital syphilis is one of the preventable infections and is easily treated, as long as testing and treatment are provided to pregnant women early during antenatal care. Infants with congenital syphilis can develop an acute systemic illness (pneumonia, non-immune hydrops), bone deformities, developmental disabilities, blindness, or deafness immediately or later in life (7). Our patient was diagnosed with congenital syphilis at 5 months of age, which represents a delayed diagnosis.

Reflecting on this patient's presentation, we identified several missed opportunities where intervention from healthcare workers could have prevented or detected and treated this condition earlier.

Although syphilis is one of the notifiable communicable diseases worldwide; there is no mandate for reporting in Lebanon. Thus, we suspect that several cases are not reported to the ministry of public health. In addition to early reporting by physicians; it is imperative to provide diagnosed patients not only with appropriate treatment but also with education about the proper strategies to inform their sexual partner(s) of the possible exposure and to avoid transmission of syphilis to them (8). The health department or the patient's physician should perform contact tracing and partner notification. Although the father was treated upon his initial diagnosis; he failed to inform the mother and her treating obstetrician. In addition, she was not tested for syphilis.

Regardless of the partner's diagnosis; the WHO and CDC recommend screening all pregnant women for syphilis at the first prenatal encounter with a repeat screening for women at high risk of infection early in the third trimester and at delivery (4, 9, 10). Pregnant women who were not screened during pregnancy should be tested at delivery (2, 4, 9, 10). This allows prompt identification and treatment of infected newborns.

Several studies show that timely detection of syphilis in pregnant women and appropriate treatment with penicillin result in a reduction in the incidence of clinical congenital syphilis by 97% (11–13).

In addition, untreated syphilis during pregnancy is a common cause of stillbirth or neonatal death (11).

The patient's mother had routine prenatal care; she had several visits throughout the pregnancy that included prenatal screening for anemia, hepatitis B infection, rubella and toxoplasma serology. Testing for syphilis was not offered to the mother. To note that in Lebanon, routine screening for syphilis is not offered to pregnant women. This could be due in part to the stigma related to sexually transmitted infections in general and syphilis in a very conservative culture.

The CDC recommends not discharging newborns from the hospital unless the syphilis serologic status of the mother has been documented at least one time during pregnancy and at delivery if at risk (14). Applying this recommendation in the hospitals would present a great opportunity to detect mothers and newborns with syphilis infection who were missed during prenatal care.

Our patient's newborn exam was reported as normal which is consistent with the fact that 60–90% of live newborns with congenital syphilis are asymptomatic at birth (15). At 2 months of age, he started to develop several signs and symptoms including failure to thrive, “snuffles,” maculopapular rash and bulging fontanelle which are significant clinical manifestations of early congenital syphilis (6, 7, 16).

Treponema pallidum can only be cultured *in vivo*; serologic tests including non-treponemal and treponemal antibodies remain an important tool for the diagnosis of syphilis (17). In this reported case, the initial VDRL test was falsely negative probably due to the prozone phenomenon, which can happen during any phase of syphilis. It occurs when a non-treponemal test is performed on undiluted serum samples that contain a high concentration of *Treponema pallidum* antibodies, leading to the blockage of antigens and interfering with the antigen-antibody lattice formation, resulting in a weakly reactive or falsely negative result. Serum dilution is recommended as a routine to prevent this phenomenon (17, 18). The prozone phenomenon should be taken into consideration by providers in any infant with suspected congenital syphilis.

In conclusion, congenital syphilis can be prevented by using basic public health measures as demonstrated in several low and middle income countries (9). Appropriate prenatal care with syphilis serologic testing at the first prenatal visit of all pregnant women regardless of their risk factors should be mandatory even in countries with conservative cultures. In addition, as per the CDC recommendations, no mother and/or newborn should be allowed to leave the hospital without documenting the maternal serologic status for syphilis at least once (6). Finally, educational interventions targeted toward healthcare workers need to address the early diagnosis of syphilis/congenital syphilis, reporting to the health authorities, and providing education regarding prevention and treatment to patients and their sexual partners.

## Data Availability Statement

The datasets generated for this study are available on request to the corresponding author.

## Ethics Statement

A written informed consent was obtained from the patient's father for the publication of this case report.

## Author Contributions

All authors listed have made a substantial, direct and intellectual contribution to the work, and approved it for publication.

## Conflict of Interest

The authors declare that the research was conducted in the absence of any commercial or financial relationships that could be construed as a potential conflict of interest.
